# Age-related trends in anti-Mullerian hormone serum level in women with unilateral and bilateral ovarian endometriomas prior to surgery

**DOI:** 10.1186/s12958-015-0125-x

**Published:** 2015-11-24

**Authors:** Dorota Nieweglowska, Iwona Hajdyla-Banas, Kazimierz Pitynski, Tomasz Banas, Oliwia Grabowska, Grzegorz Juszczyk, Artur Ludwin, Robert Jach

**Affiliations:** Department of Gynecology and Oncology, Jagiellonian University, Chair of Gynecology and Obstetrics, Krakow, 21 Kopernika Str, 30-501 Krakow, Poland; Center of Rheumatology, Immunology and Rehabilitation, Dietl Specialistic Hospital, Krakow, Poland; Nuffield Division of Clinical Laboratory Science, University of Oxford, Level 4, John Radcliffe Hospital, Headington, OX3 9DU Oxford, UK; Department of Public Health, Medical University of Warsaw, Warsaw, Poland; Department of Gynecological Endocrinology, Jagiellonian University Medical College, Krakow, Poland

**Keywords:** Anti-Mullerian hormone, AMH, Ovarian endometrioma, Endometriosis

## Abstract

**Background:**

Endometriosis is a well-known cause of infertility, and the anti-Mullerian hormone (AMH) is an accepted biomarker of ovarian reserve and response to artificial reproductive technology procedures. The present study was a prospective analysis of age-dependent AMH serum concentration in women with bilateral and unilateral ovarian endometriomas before therapy onset compared with healthy controls.

**Methods:**

This prospective cross-sectional study included 384 women aged 18–48 years. AMH serum concentration was assessed between days 3 and 6 of the menstrual cycle in 78 patients with bilateral and 157 patients with unilateral ovarian endometriomas and compared with 149 healthy controls. Ovarian endometriosis was confirmed histopathologically, and data were presented as medians with interquartile range (IQR).

**Results:**

Stage III endometriosis was diagnosed in 53.2 %, stage IV in 18.3 %, stage V in 23.4 % and stage VI in 5.4 % of the patients. Patients with bilateral ovarian endometriomas showed the lowest median AMH levels compared with patients suffering from unilateral ovarian endometriosis (0.55; IQR: 0.59 *vs.* 2.00; IQR: 2.80; *p* < 0.001) and the control group (0.55; IQR: 0.59 *vs.* 2.84; IQR: 3.2; *p* < 0.001). Median AMH concentration values were not significantly different between patients with unilateral ovarian endometriosis and the healthy controls (2.00; IQR: 2.80 *vs.* 2.84; IQR: 3.2; *p* = 0.182). A strongly negative correlation between AMH levels and age was confirmed in healthy individuals (R = −0.834; *p* < 0.001) and women with unilateral ovarian endometriomas (R = −0.774; *p* < 0.001). Patients with bilateral ovarian endometriosis showed a significantly negative but only moderate correlation between AMH levels and age (R = −0.633; *p* < 0.001), which was significantly lower than in the healthy controls (R = −0.633 *vs.* R = −0.834; *p* = 0.006) but not in the patients with unilateral ovarian endometriosis (R = −0.663 *vs.* R-0.774; *p* = 0.093). Based on a multivariate regression analysis, only bilateral localization of ovarian endometrial cysts (*p* = 0.003) and patient age (*p* < 0.001), but not left/right localization of unilateral cyst or cyst volume, were negatively associated with AMH serum concentration.

**Conclusion:**

According to our data, unilateral ovarian endometriosis had a moderately negative and nonsignificant effect on AMH-based ovarian reserve evaluated prior to surgery, irrespective of age. In contrast, the ovarian reserve was significantly reduced in women with bilateral ovarian endometriomas.

## Background

Infertility is an increasing medical and socioeconomic problem affecting up to 15 % of couples [1]. Fortunately, many of these patients have an opportunity for parenting, due to rapidly developing diagnostics and artificial reproduction techniques (ARTs). Among many factors responsible for fertility problems, endometriosis is often recognized in women diagnosed with infertility [2].

Endometriosis affects up to 10 % of women of reproductive age, and its pathogenesis, despite numerous studies, has not been elucidated [3]. As many as 40 % of patients with endometriosis will face fertility problems [4], which can be caused by direct ovarian destruction, impotence of fallopian tubes, due to intraperitoneal adhesions or impaired follicle growth, and ovulation disorders resulting from local pelvic inflammation [5]. Women diagnosed with endometriosis suffer from endometrial polyps and have a high rate of implantation failure [6]. Moreover, endometriomas may damage otherwise healthy ovarian tissue. Hughesdon described the ovarian cortex near an endometrioma as stretched and disorganized with evidence of smooth muscle metaplasia [7]. Recent studies have identified several toxic agents, such as pro-inflammatory cytokines, reactive oxygen species (ROS) and iron deposits in endometriotic fluid [8, 9]. Because the barrier separating cyst fluid from normal ovarian tissue is 1 mm thick and composed of fibroreactive tissue and the ovarian cortex, endometriotic fluid is thought to be highly harmful to the surrounding cells and the healthy ovarian cortex tissue near the endometrioma. ROS and cytokines cause fibrosis of the ovarian tissue and a reduction in cortex-specific stromal cells [9]. Subsequently, the fibrosis together with the ROS-triggered decrease in angiogenesis and capillary loss in the ovarian cortex impair follicle nutrition and may be responsible for lower follicular density and functional follicle loss in the ovaries with endometriosis [10]. These findings support the theory that endometriomas cause ovarian damage before surgical treatment.

Many women with pelvic endometriosis and concomitant infertility require ART procedures. However, patients suffering from endometriosis have also been reported to show a decreased ovarian reserve compared to healthy individuals of the same age [11, 12]. The evidence of endometriosis was also proven to be a risk factor for a reduced response to controlled ovarian stimulation in women undergoing ART [13]. The anti-Mullerian hormone level is a useful marker of the ovarian reserve and response used in fertility therapy [14] and allows individualized treatment, reducing clinical risk of ART-related complications and improving the pregnancy rate [15].

AMH was identified as a glycoprotein dimer composed of two monomers of 72 kDa, each connected with disulfide bridges and belongs to the transforming growth factor β (TNFβ) family [16]. AMH is produced exclusively by granulosa cells of ovarian follicles and may have a regulatory effect on the axial folliculogenesis [17]. Experiments in mice suggest that AMH inhibits the growth of primary follicles and is involved in the growth regulation of antral, preantral and small follicles by inhibiting their sensitivity to follicle-stimulating hormone (FSH) [18]. AMH secretion is strongly associated with age, with production starting in the 36^th^ week of fetal life and reaching a peak in puberty before continuously decreasing until menopause when often reaches undetectable values [19, 20]. Many studies confirmed that AMH levels decline with age in peripartum and in patients with severe pelvic edometriosis but remains stable during the menstrual cycle, which allows for its random determination irrespective of the cycle day [17, 21–24]. However, Hadlow et al. reported variations in AMH serum concentration during the menstrual cycle with a gradual decrease from the early-follicular to the late-luteal phase of the menstrual cycle with peak values in the mid-follicular phase [25].

AMH is an accepted biomarker of the ovarian reserve and response to ART procedures; however, a lack of reference values established separately for female cohorts with different ovarian pathology can be a major drawback in its clinical utility. Based on our literature review, we hypothesize that endometriomas themselves can impair ovarian reserve irrespective of surgical treatment. To clear this hypothesis, we set up a prospective study evaluating AMH levels in women with ovarian endometriosis. The aim of the study was a preoperative evaluation of serum AMH concentration in women with unilateral and bilateral endometriomas, depending of the ages of the patients.

## Methods

All women referred to the Gynecology and Oncology Department and Endocrine Gynecology Department of the Jagiellonian University Medical College, Krakow, from January 2009 to December 2014, were constructively and prospectively evaluated for this study. Inclusion criteria were age (18–48 years) and bilateral or unilateral ovarian endometriosis diagnosed during laparoscopy or laparotomy and confirmed histopathologically. We excluded the following subjects: (1) pregnant women; (2) patients with previous excision of ovarian cysts; (3) patients diagnosed with infertility (unless solely related to endometriosis or the male factor infertility); (4) patients who had received hormonal treatment during the prior 36 months; (5) patients diagnosed with endocrine disorders; (6) patients suffering from chronic disease (defined as illness lasting 3 months or more, according to the U.S. National Center for Health Statistics); and (7) patients with a history of malignancy. The control group consisted of healthy volunteers who participated in the National Screening Program against Cervical Cancer. Control group exclusion criteria included a history of (1) infertility, (2) endometriosis, (3) early pregnancy loss, (4) hormonal disorders, (5) malignancy, and (6) pelvic/abdominal cavity surgery. All the participating women signed an informed consent document, and the research was approved by the Jagiellonian University Ethics Board (KBET/21/B/2009).

### Diagnostic procedures

Every woman meeting the inclusion criteria had her medical history recorded and underwent a bimanual pelvic examination by an experienced gynecology or gynecologic endocrinology consultant. Subsequently, a transvaginal ultrasonography was performed using a Voluson 730 Pro equipped with a 6.5 MHz trans-vaginal probe (General Electric Medical Systems, Kretztechnik, Zipf, Austria) to evaluate the uterus and ovaries and look for the presence of endometrial cysts. The volume of each endometrial cyst was evaluated in every case and expressed in cm^3^.

### Surgical treatment

The majority of the patients (98.3 %) with an initial diagnosis of endometrial ovarian cysts underwent routine unilateral/bilateral laparoscopic cyst enucleation and excision of peritoneal endometriosis. In three cases, a conversion to laparotomy was performed due to severe intraoperative bleeding (*n* = 1) and the need for partial large bowel resection and anastomosis (*n* = 3). None of the patients required hysterectomy or adnexectomy. The bleeding was controlled by bipolar coagulation (85.5 %) and ovarian suturing (14.5 %).

Histopathological examination: Although histopathological evaluation of endometriosis visualized during surgery was not necessary to make the final diagnosis, all of the excised specimens were examined postoperatively by experienced pathologists using hematoxylin-eosin staining and immunohistochemistry, according to our routine procedures. The postoperative diagnosis was based on the final histopathological report confirming ovarian endometriosis.

### Blood analysis

Venous blood samples were analyzed between days 3 and 6 of the menstrual cycle to determine the AMH levels. In patients with ovarian endometriomas, the average time between blood collection and surgery was 33 days (range 3–47 days) as the vast majority of women were scheduled for operation during the next menstrual cycle after blood collection. Immediately after collection, the samples were centrifuged for 15 min at 1400 rpm. The serum was aspirated and transferred to 1.5 mL Eppendorf tubes and stored for up to 3 months at −80 °C. Baseline blood samples were collected between days 3 and 7 of the menstrual cycle. The biochemical analysis was performed using the enzyme-linked immunosorbent assay (ELISA) method. The 96-well plates (Biokom) were incubated with the blood serum (50 μL per well) for 12 h at 4 °C. Each plate was washed with 3 x 200 μL PBS (pH 7.0) using an automatic scrubber. The plates were re-incubated with the primary antibody at a dilution of 1:10,000 (Thermo Scientific) for 12 h at +4 °C. Next, the plates were incubated for 2 h with secondary antibody labeled with horseradish peroxidase (HRP) and a 15 min incubation with 3,3′,5,5′-tetramethylbenzidine (TMB, Thermo Scientific). Immediately after putting on the stop solution (1 nM HCl, Thermo Scientific), the plates were read using an ELISA reader with KC Junior program. Measurements were performed in duplicate. Standardization curves included 8 steps at the stage r = 0.95. The serum AMH concentration was expressed in ng/mL, and the functional detection limits ranged from 0.05 to 15.00 ng/mL.

### Statistical analyses

The Kolmogorov-Smirnov test was used to evaluate the distribution of variables. To compare the variables with normal distribution, an analysis of variance (ANOVA) test was performed, and the data were presented as the mean values with standard deviation (SD). To analyze continuous data with a different distribution than normal and quantitive variables, a chi square test or Kruskal-Wallis ANOVA was used, and baseline characteristics were presented as median values with interquartile range (IQR). Post hoc exploration (Fisher’s least significant difference test) was performed if significant differences were found during the ANOVA analysis. Multiple regression was used to analyze the impact of different clinical variables on the serum AMH level. Because serum AMH concentration showed a skewed distribution, logarithmic transformation was applied for further testing. Spearman’s correlation test was used to analyze the relationship between AMH serum level and age, and the results were presented using Spearman’s correlation index (R). A *p*-value of 0.05 was considered to indicate statistical significance. All calculations were performed using STATISTICA data analysis software, version 10.0 (StatSoft, Inc. 2011, Tulsa, OK, USA).

## Results

**Fig. 1:**
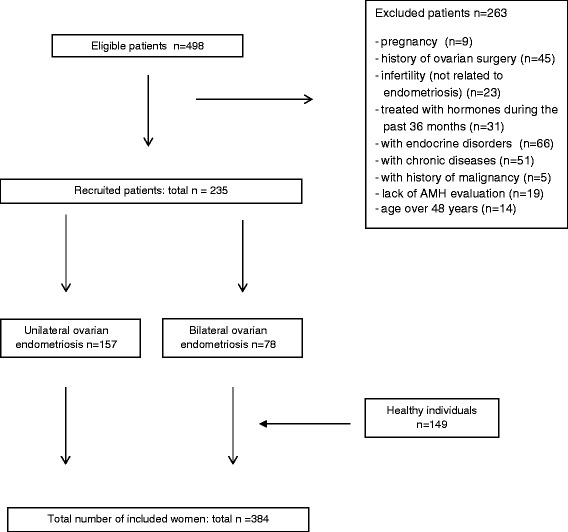
Flow diagram demonstrating the patient recruitment procedure

A total of 498 women were eligible for the study, and 235 fulfilled the inclusion criteria (78 women with bilateral and 157 with unilateral and ovarian endometriomas; Fig. 1). Among the patients with endometriosis, 68 were part of a couple experiencing fertility problems, 86 had pelvic pain syndrome (PPS), and 91 were diagnosed with ovarian endometriosis during a routine gynecological examination and referred for further management. Male factor infertility was diagnosed in 17 of the 68 infertile pairs. In 51 couples, a comprehensive infertility examination revealed no potential causes of infertility apart from the presence of endometriomas. These women were diagnosed with endometriosis-related infertility and included in the study. The control group consisted of 149 women with no gynecological disorders, a history of pelvic/abdominal surgery, pregnancy or chronic diseases and who attended cervical cancer screening programs.

More than half of the patients (53.2 %) suffering from endometriosis were diagnosed with stage III disease according to the European Society of Human Reproduction and Embryology (ESHRE) criteria [26]. Stage IV endometriosis was recognized in 18.3 % of women, and stages V and VI were confirmed in 23.4 % and 51 %, respectively. There were no cases of stage I-II endometriosis because such patients do not present with an ovarian mass. In patients with unilaterality, ovarian endometrial cysts were predominantly localized in the right ovary (Table 1). There were no differences in ovarian endometrioma volume between patients with bilateral and unilateral ovarian mass (Table 1). Women with bilateral and unilateral ovarian endometriosis suffered significantly more frequently with painful and irregular menstruation compared to healthy women (Table 1). Patients suffering from bilateral but not unilateral ovarian endometriomas presented significantly lower gravidity (*p* < 0.001) and parity (*p* = 0.003) compared to the control group. No significant differences in median age, median age of first period, body mass index (BMI) or amount of menstrual discharge were noticed between the groups analyzed (Table 1).

**Table 1:** The clinical characteristics of patients with bilateral and unilateral ovarian endometrioas and the healthy women

	Patients with bilateral ovarian endometrimas (*n* = 78)	Patients with unilateral ovarian endometrimas (*n* = 157)	Healthy controls (*n* = 149)	*p*
Median age [yers] (IQR^b^)	35,50 (17,00)	32,00 (15,00)	32,00 (18,00)	NS^c^
Mean BMI [kg/m^2^] (±SD^a^)	22,72 (±1,61)	23,19 (±2,13)	23,41 (±2,51)	NS^c^
Endometrial cyst localization				
Right ovary	78 (100,00 %)	93 (59,23 %)	NA^e^	NA^e^
Left ovary	78 (100,00 %)	64 (40,77 %)		
Mean volume of ovarian endometriomas [cm^3^]	6,23 (±1,12)	6,41 (±1,18)	NA^e^	NS^c^
Mean age of first menstrual period [years] (±SD^a^)	11,52 (±1,52)	11,38 (±1,64)	11,45 (±1,81)	NS^c^
Mean duration of menstrual cycle [days] (±SD^a^)	28,50 (±2,5)	29,50 (±3,0)	28,00 (±2,0)	NS^c^
Menstrual cycles	42 (53,85 %) /	116 (73,89 %) /	124 (83,22) /	<0,001^d^
Regular/Irregular	36 (46,15 %)	41 (26,11 %)	25 (16,78 %)	
Menstrual cycles	38 (48,72) /	42 (26,75 %) /	32 (21,48) /	<0,001^d^
Painful/painless	40 (51,28 %)	115 (73,25 %)	117 (78,52 %)	
Mean duration of menstruation [days]	4,25 (±1,52)	4,12 (±1,31)	4,32 (±0,97)	NS^c^
Type of menstrual bleeding				NS^c^
Scant	7 (11,54 %)	12 (7,64 %)	8 (10,69 %)	
Normal	52 (66,67 %)	115 (73,25 %)	117 (75,52 %)	
Heavy	17 (21,79 %)	30 (19,11 %)	24 (13,79 %)	
Median number of gestations (IQR^b^)	1,0 (1,0)	2 (2)	3 (2)	0,028^d^
Median number of deliveries (IQR^b^)	0,5 (1)	2 (1)	3 (1)	0,016^d^

^a^SD – standard deviation; ^b^IQR – interquarlite range; ^c^NS – statistically not significant; ^d^statistically significant value; ^e^NA – data not available

Patients with bilateral ovarian endometriomas presented the lowest median AMH levels, compared to women suffering from unilateral ovarian endometriosis (0.55; IQR: 0.59 *vs.* 2.00; IQR: 2.80; *p* < 0,001) and the control group (0.55; IQR: 0.59 *vs.* 2.84; IQR: 3.2; *p* < 0.001). Patients with ovarian unilateral endometriosis also showed lower but insignificant median AMH levels compared to the control group (2.00; IQR: 2.80 *vs.* 2.84; IQR: 3.2; *p* = 0.182). Based on multivariate regression, only bilateral localization of ovarian endometrial cyst (*p* = 0.003) and patient age (*p* < 0.001) but not localization or cyst volume were negatively associated with the AMH serum concentration.

**Fig. 2:**
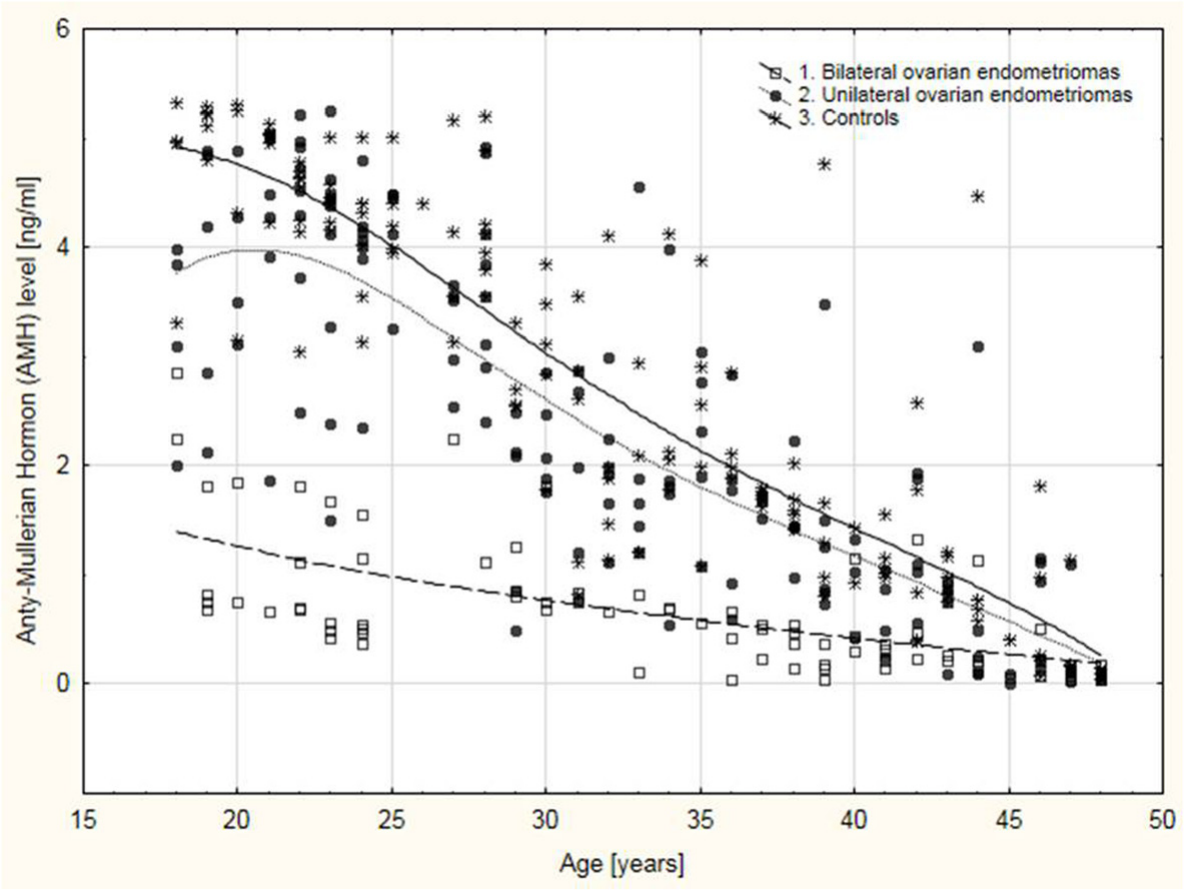
Age-related anti-Mullerian hormone (AMH) distribution in patients with bilateral ovarian endometriomas, unilateral ovarian endometriomas and the controls

An age-related decrease in AMH levels was confirmed in the control group and in patients with unilateral and bilateral ovarian endometriomas (Fig. 2).

A strong negative correlation between AMH levels and age was confirmed in the healthy controls (R = −0.834; *p* < 0.001) and women with unilateral ovarian endometriosis (R = −0.774; *p* < 0.001). In women with bilateral ovarian endometriosis, a moderately negative correlation between AMH levels and age was observed (R = −0.663; *p* < 0.001). Correlation indices for AMH levels and age did not differ significantly between women with unilateral endometriosis and the controls (R = −0.774 *vs.* R = −0.834; *p* = 0.140). However, patients with bilateral ovarian endometriomas showed a significantly weaker correlation between AMH levels and age compared to the controls (R = −0.633 *vs.* R = −0.834; *p* = 0.006) but not the patients with unilateral ovarian endometriosis (R = −0.663 *vs.* R = −0.774; *p* = 0.093).

**Fig. 3:**
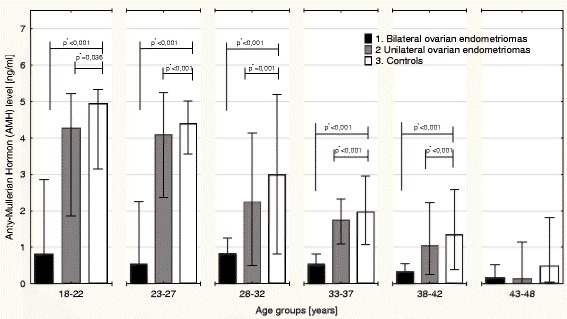
Differences in serum anti-Mullerian hormone (AMH) concentration levels between patients with bilateral and unilateral ovarian endometriomas compared to healthy individuals in different age groups. *statistical significance at *p* < 0.05

For further analysis of AMH serum level, participants were divided according to age into the following 6 groups: 18–22, 23–27, 28–32, 33–37, 38–42 and 43–48 years. Serum AMH levels were then evaluated in patients with bilateral and unilateral ovarian endometriomas and compared to healthy controls in the same age-related groups. In all age groups, serum AMH concentration was the highest in healthy controls. However, in women aged 43 years and above, no significant differences in AMH levels were observed between patients with bilateral ovarian endometriomas, unilateral ovarian endometriomas and controls. In young women aged 18–22 years, we observed significantly lower median AMH levels in patients with bilateral ovarian endometriomas compared to the controls (0.82; IQR: 1.12 *vs.* 4.63; IQR: 1.09; *p* < 0.001) and between patients with bilateral ovarian endometriosis and unilateral ovarian endometriosis (0.82; IQR: 1.12 *vs.* 4.24; IQR: 1.24; *p* = 0.036), while differences were not observed between unilateral ovarian endometriosis patients and the controls (4.24; IQR: 1.12 *vs.* 4.63; IQR: 1.09; *p* < 0.001; Fig. 3). Additionally, women aged 23–27 years with bilateral ovarian endometriomas showed significantly lower median serum AMH concentrations compared to patients with unilateral ovarian endometriomas (0.55; IQR: 1.07 *vs.* 4.10; IQR: 1.17; *p* < 0.001) and to controls (0.55; IQR: 1.07 *vs.* 4.24; IQR: 0.44; *p* < 0.001), while no significant differences in median AMH levels were found between unilateral ovarian endometrioma patients and the healthy controls (Fig. 3). Similar to previous groups in women aged 28–32 years, patients with bilateral ovarian endometriomas showed the lowest median AMH serum levels compared to women with unilateral ovarian involvement (0.95; IQR: 0.37 *vs.* 2.25; IQR: 1.02; *p* = 0.001) and healthy controls (0.95; IQR: 0.37 *vs.* 3.00; IQR: 1.86; *p* < 0.001), with no significant differences observed between unilateral ovarian endometriomas and the healthy controls (Fig. 3). In women aged 33–37 years, the lowest median AMH concentration was observed in patients with bilateral ovarian endometriomas compared to patients with unilateral ovarian endometriomas (0.49; IQR: 0.45 *vs.* 1.75; IQR: 0.38; *p* < 0.001) and controls (0.49; IQR: 0.45 *vs.* 1.98; IQR: 0.84; *p* < 0.001; Fig. 3). Additionally, in women aged 38–42 years, the significantly lowest median serum AMH concentration was in patients with bilateral ovarian endometriomas compared to patients with unilateral ovarian endometriomas (0.35; IQR: 0.30 *vs.* 1.05; IQR: 0.70; *p* < 0.001) and the controls (0.35; IQR: 0.30 *vs.* 1.35; IQR: 0.90; *p* < 0.001; Fig. 3). In contrast to younger patients, women aged 43–48 years showed no statistically significant differences in the median AMH concentration between women with bilateral ovarian endometriomas (0.18; IQR: 0.19), unilateral ovarian endometriomas (0.14; IQR: 0.78) and the controls (0.49; IQR: 0.8; Fig. 3).

## Discussion

In our study, we confirmed a significant age-related AMH decrease in healthy women and patients with ovarian endometriomas, both bilateral and unilateral, before the onset of surgical therapy [27–30]. Patients with unilateral ovarian endometriosis showed a significantly negative correlation between serum AMH level and age. Correlation indices were not significantly different between these patients and the healthy controls. On the contrary, patients with bilateral endometriomas showed a significantly weaker correlation between AMH levels and age, which differed significantly from patients with unilateral endometriomas. Moreover, patients under 43 years of age with bilateral ovarian endometriosis showed significantly lower serum AMH concentration compared to patients with unilateral ovarian endometriosis and healthy controls. The vast majority of our participants were diagnosed with moderate endometriosis, and only a few with a severe disease. Interestingly Shebl et al. reported significant differences in AMH levels between patients with severe endometriosis and healthy individuals, while patients with mild endometriosis showed AMH serum concentration comparable to healthy controls [27]. Similar to our findings, Somigliana et al., showed that only patients with bilateral ovarian benign tumors (of which 72 % were endometrial cysts) had significantly lower AMH serum levels when compared to patients with unilateral ovarian cysts and healthy women [31]. These results were again confirmed in other studies showing no differences in serum AMH levels between patients with mild endometriosis and healthy women [32–34]. Thus, unilateral endometriosis negatively affects ovarian reserve although not to statistical significance, while bilateral ovarian endometriomas result in significantly decreased AMH levels from an early age. In women with unilateral ovarian endometrial cysts, procreative ovarian function is sustained and comparable to that of healthy individuals.

Lind et al. investigated AMH levels in women who underwent surgery for benign ovarian tumors and found that the reduction in AMH levels depended on the histological type of the ovarian cyst and preoperative AMH levels [35]. Somigalina et al. reported that presurgical AMH serum concentrations were higher in patients with dermoid cysts than in women with endometriomas; however, the difference was not statistically significant [31]. These clinical findings are consistent with the pathology of endometriotic and non-endometriotic ovarian cysts. Neither dermoid cysts nor simple ovarian cysts cause local inflammation. Thus, healthy ovarian cortical tissue surrounding a non-endometriotic cyst is not exposed to pro-inflammatory agents or ROS, which are present in endometriotic fluid. Furthermore, in contrast to endometriomas, non-endometriotic ovarian cysts do not contain iron deposits, which cause the hemosiderin-laden macrophages that trigger follicular destruction in the surrounding cortical tissue. Thus, it appears that dermoid and simple ovarian cysts do not directly impair ovarian function. Nevertheless, it must be emphasized that surgical intervention and ovarian cystectomy for benign ovarian tumors are independent risk factors for reduced ovarian reserves.

Ovarian endometrial cysts may be directly responsible for a decrease in AMH levels. However, surgical treatment of endometriomas may be an additional and independent risk factor for impaired ovarian function. Several studies have investigated the impact of surgery on ovarian function in women with endometriosis. Interestingly, Streuli et al. reported that decreased serum AMH levels in women with endometriosis were limited only to those with previous endometrioma surgery [36]. Moreover, in patients undergoing laparoscopic cystectomy due to ovarian endometriosis, a significant reduction in AMH secretion was reported [33]. Women with higher preoperative AMH levels showed a more rapid decrease in AMH levels. Similarly, in patients with unilateral ovarian endometrioma, the decrease in AMH levels was more significant and lasted longer compared to women who underwent surgery for dermoid cysts [35]. In contrast, Vignali et al. reported that the postoperative decrease in AMH levels in women who had undergone laparoscopic excision of ovarian endometrial cysts was temporary; AMH levels returned to preoperative values 12 months after surgery [37]. Angioni et al. investigated the feasibility of single-port access laparoscopy (SPAL) compared with multiport laparoscopy (MPL) for cystectomy of ovarian endometriomas [38]. Over a 3-month follow-up period, they observed a significant decrease in AMH serum concentrations and antral follicle count after SPAL compared with MPL and concluded that SPAL cystectomy should not be recommended for women with endometriomas who desire pregnancy [38]. However, in addition to surgical access, the cyst extirpation method and bleeding control can influence ovarian reserve. Although excisional surgery is the gold standard treatment for endometriotic cysts, it may result in unintended removal of healthy ovarian tissue. Moreover, the use of bipolar coagulation to control bleeding may damage healthy ovarian tissue, causing a decrease in ovarian reserve. Nappi et al. found that laser hemostasis using a dual-wavelength system did not significantly reduce ovarian reserve and prevented follicular loss after endometrioma surgery [39]. Thus, laser hemostasis may be a better choice than bipolar coagulation for women with endometriomas who wish to preserve fertility. Many studies were in accordance with our findings that unilateral ovarian endometriosis, which is considered to be a moderate endometriosis according to ESHRE criteria, does not impair ovarian procreation function at any age.

As the procreative function of ovaries declines rapidly after 35 years of age, we analyzed the association between age and AMH levels after stratification of patients according to age. Serum AMH levels were significantly lower in women with bilateral ovarian endometriosis who were under 43 years of age than in age-matched patients with unilateral endometriomas and healthy individuals. However, in women over the age of 42 years, no differences in median serum AMH concentration was observed between patients with bilateral or unilateral ovarian endometriosis and the controls. This result can be explained because after the age of 40 years, ovarian follicle function has decreased to the extent that endometriosis does not further reduce ovarian reserve.

To our knowledge, this is the first comprehensive study evaluating the relationship between serum AMH concentration levels and age in women with bilateral and unilateral ovarian endometriomas prior to surgery. All laboratory evaluations were performed in one setting by staff highly experienced in blood collection for serum AMH level assessment, performed at a narrow point in time to reduce research bias. However, this study had several limitations. The primary limitation was the low number of patients with bilateral ovarian endometriomas. Second, women with incidental endometriomas and PPS who experienced irregular periods were not evaluated to identify the cause of menstruation irregularity. Thus, it is possible that undiagnosed disorders were the cause of impaired ovarian function in these women. However, this potential bias was significantly mitigated by an extensive review of available medical records and histories to detect risk factors for impaired ovarian function apart from endometriosis, although the presence of an undiagnosed disorder could not be completely ruled out. The results obtained should be externally validated in a larger cohort to gain an epidemiological impact. Only a prospective follow-up of AMH secretion in women with ovarian endometriomas conducted in a large population and with age-matched controls over a longer period of time will allow us to fully elucidate its clinical utility.

## Conclusions

In the present study, we showed a significantly negative correlation between serum AMH concentration and age in women with bilateral and unilateral ovarian endometriosis. However, median serum AMH levels were significantly lower only in patients with bilateral ovarian endometriomas compared to controls, while in patients with unilateral ovarian endometriomas, median serum AMH concentration was insignificantly lower compared to the healthy individuals. According to our data, unilateral ovarian endometriosis moderately impaired AMH-based ovarian reserve prior to surgery, irrespective of age. In contrast, in women with bilateral ovarian endometriomas, a significant reduction in ovarian reserve was shown.

## Abbreviations

ANOVA: analysis of variance
AMH: anti-mullerian hormone
ART: artificial reproductive technology
ELISA: enzyme-linked immunosorbent assay
FSH: follicle-stimulating hormone
IQR: interquartile range
NS: statistically not significant
NA: data not available
TNF: tumor necrosis factor
NK: natural killer cells
R: correlation index
SD: standard deviation
